# Elastic–Plastic Analysis of Asperity Based on Wave Function

**DOI:** 10.3390/ma18153507

**Published:** 2025-07-26

**Authors:** Zijian Xu, Min Zhu, Wenjuan Wang, Ming Guo, Shengao Wang, Xiaohan Lu, Ziwei Li

**Affiliations:** Naval Engineering University, Wuhan 430033, China; 18153567179@163.com (Z.X.); morpheusgwo@163.com (M.G.); wangshengao123@163.com (S.W.); lxh13793754591@163.com (X.L.);

**Keywords:** wavy asperity, hyperbolic tangent function, finite element method

## Abstract

This paper proposes an improved wave function asperity elastic–plastic model. A cosine function that could better fit the geometric morphology was selected to construct the asperity, the elastic phase was controlled by the Hertz contact theory, the elastoplastic transition phase was corrected by the hyperbolic tangent function, and the fully plastic phase was improved by the projected area theory. The model broke through the limitations of the spherical assumption and was able to capture the stress concentration and plastic flow phenomena. The results show that the contact pressure in the elastic phase was 22% higher than that of the spherical shape, the plastic strain in the elastoplastic phase was 52% lower than that of the spherical shape, and the fully plastic phase reduced the contact area error by 20%. The improved hyperbolic tangent function eliminated the unphysical oscillation phenomenon in the elastoplastic phase and ensured the continuity and monotonicity of the contact variables, with an error of <5% from the finite element analysis. Meanwhile, extending the proposed model, we developed a rough surface contact model, and it was verified that the wavy asperity could better match the mechanical properties of the real rough surface and exhibited progressive stiffness reduction during the plastic flow process. The model in this paper can provide a theoretical basis for predicting stress distribution, plastic evolution, and multi-scale mechanical behavior in the connection interface.

## 1. Introduction

Rough surfaces of connected structures include a variety of contact mechanisms, such as friction, wear, sealing, etc., all of which are complex, nonlinear mechanical behaviors [1]. The study of such phenomena has a crucial position in the fields of tribology, materials science, and mechanical engineering and is a hot topic at present. The research paradigm using the assumption of asperities has been established since the landmark statistical contact model (GW model) proposed by Greenwood and Williamson [2]. The GW model assumes a rough surface as a series of spherical asperities with the same radius of curvature, and the contact behavior of the asperities obeys Hertz’s theory of elastic contact, but the assumption does not consider the elastic and plastic states; therefore, it is not possible to deal with the actual contact behavior of rough surfaces. As this assumption does not consider the elastic–plastic and plastic states, the limitations gradually appear when dealing with elastic–plastic deformation and multiscale effects on real engineering surfaces, and the theoretical analysis error increases with increases in contact pressure.

In order to more accurately describe the elastic–plastic contact process, the focus of subsequent research has been on improving the mechanical model of asperities. The CEB model proposed by Chang, Etsion, and Bogy [3] extends the GW model by systematically considering, for the first time, the continuous deformation process of asperities from elasticity to plasticity. The model establishes the contact load–area relationship in the elastic–plastic phase by defining the critical contact depth $\omega_{c}$ and the fully plastic contact depth $\omega_{p}$. However, the CEB model has discontinuous contact variables at the critical yield point $\omega_{c}$ and jumps in the elastic–plastic curve, which is contrary to the physical law of real contact.

To solve the discontinuity problem of the CEB model at the critical point, Chang et al. [4] proposed an improved CEB model; however, it only considered the fully elastic and fully plastic contact states and did not analyze the elastic–plastic phase in depth. Abbott et al. [5] proposed that after the plastic yielding of the asperity occurs, the pressure on the contact surfaces and the hardness of the material, H, are in a linear relationship. Then, the contact surface obeys the assumption of equal area, the asperity obeys the assumption of volume conservation, and the AF model for the fully plastic phase is obtained. Zhao et al. [6,7] proposed the ZMC model on the basis of this model, which connects the elastic solution with the plastic solution by using a high-degree polynomial interpolation method, which ensures the continuity of the contact variables at $\omega_{e}$, but its introduction of the high-order interpolation term leads to nonphysical oscillations of the contact pressure in the elastic–plastic phase. Kougut et al. [8] concluded that the critical points of the first and second elastic-plasticity also exist in the elastoplastic phase through finite element analysis, and therefore, the nonlinear relationship of the elastic–plastic phase is satisfied by the segmented function description in combination with the plasticity index, so the KE model is established. Brake [9] used Hermit’s interpolation function to ensure the continuity and smoothness at two critical points of the elastic–elastoplastic phase and elastoplastic–plastic phase and the Brake model was established; however, this also led to the occurrence of the Runge phenomenon [10] (oscillations at the edges of high-degree polynomial interpolation), and the solution of the equation was not unique.

In recent years, the optimization of asperity geometry itself has become a key direction to improve the prediction ability of models. Chao Xu et al. [11,12] proposed various innovative ideas to improve the continuity of the model, which abandoned the high-degree polynomial interpolation and adopted elliptic curves, power functions, etc., and used low-order functions to realize the strictly monotonous and continuous change in the contact variables in the elastoplastic phase, which effectively avoids the oscillatory problem of the ZMC model. However, the surface morphology of the asperity is still based on the simplified assumption that the asperity is spherical, which is convenient for mathematical processing but difficult when trying to portray the multi-scale, periodic, and asymmetric features that widely exist on real rough surfaces. The above models all use the geometric assumption of spherical asperity. Chu et al. [13] found that the wave function has a significant advantage over the traditional model in describing the contact pressure distribution and the real contact area of the rough surface, and its prediction is more consistent with experimental observation when large deformation and plastic flow are involved; however, the elastic–plastic model used for a single asperity is the JG model [14], which still assumes spherical geometry.

This paper builds upon the aforementioned research to conduct an in-depth exploration. Leveraging the advantages of wave functions in geometric realism, the cosine function was selected to describe the morphology of a single asperity, and a new theoretical model for a wave-shaped asperity was constructed. Compared to the theoretical model of a spherical asperity, this model exhibits superior mechanical response characteristics in the elastic, elastoplastic, and plastic phases, resolves continuity and oscillation issues using low-order functions, and is applicable to contact problems involving rough surfaces.

## 2. Methodology

### 2.1. Elastic–Plastic Contact Model of Wavy Asperity

Due to the influence of processing techniques on the rough surface morphology and the experimental conditions of surface morphology measurement, this paper innovatively chooses the cosine function to construct the surface profile of a single asperity, that is, x=0 is the highest point of the asperity, and because the asperity in the three-dimensional structure, it presents axisymmetric characteristics, so its mechanical properties with the radial distribution are the same. To facilitate the analysis and calculation, this paper selects an asperity in the xoy plane for modeling. In order to facilitate the analysis and calculation, an asperity in the xoy plane is chosen for modeling in this paper:

(1) $$y=f\left(x\right)=h\cos\left(\frac{\pi x}{2L}\right)\;\;\;(-L\leq x\leq L)$$

where h is the height of the asperity, L is the radius of the wavy asperity, i.e., 1/4 of the wavelength of the single-period cosine function, the asperity is axisymmetric, the elastic-elastoplastic and the elastoplastic-plastic critical points of asperity refer to the calibration of the CEB model, which is currently recognized by most scholars.

#### 2.1.1. Elastic Phase $\left(\omega \leq \omega_{e}\right)$

First, the definition of the elastic–elastoplastic demarcation point is carried out. According to the Brake model [9] for the study and analysis of asperities, the critical contact depth $\omega_{c}={\left(\frac{3\pi KH}{4E^{*}}\right)}^{2}R$, where K=0.454+0.41ν is the plasticity correction factor [15], υ is Poisson’s ratio of the asperity, $E^{*}$ is the equivalent modulus of elasticity $\frac{1}{E^{*}}=\frac{1-{\upsilon_{1}}^{2}}{E_{1}}+\frac{1-{\upsilon_{2}}^{2}}{E_{2}}$, where $E_{1}$, $E_{2}$ and $\upsilon_{1}$, $\upsilon_{2}$ are Young’s moduli and Poisson’s ratios of the two materials, respectively, the elastic contact depth [6] $\omega_{e}\leq \omega_{c}$, the fully plastic contact depth $\omega_{p}\geq 110\omega_{c}$, $\omega_{c}$ is the critical elastic contact depth, H is the hardness of the material, where H=2.8Y [15], Y is the yield strength, and R is the contact radius of the asperity, regardless of the influence of processing technology and plastic hardening.

According to the elastic contact assumption and under the small deformation assumption, the contact region is approximated as a flat plate in contact with a wave function asperity [2], so it is necessary to solve for the radius of curvature of the function at the vertex at x=0. According to the two-dimensional morphology function f(x) of the asperity, the first-order derivative and second-order derivative are calculated as follows:

(2) $$f^{\prime }(x)=-\frac{\pi h}{2L}\sin\left(\frac{\pi x}{2L}\right)$$

(3) $$f^{\prime \prime }(x)=-\frac{\pi^{2}h}{4L^{2}}\cos\left(\frac{\pi x}{2L}\right)$$

According to the radius of curvature, $R=\frac{1}{k}$, where k is the curvature, defined according to the mathematical formula for curvature:

(4) $$k=\frac{\left|f^{\prime \prime }(x)\right|}{{\left(1+f^{\prime }{\left(x\right)}^{2}\right)}^{3/2}}$$

The radius of curvature at the contact point x=0 at the top of this cosine function is obtained as $R=\frac{1}{k}=\frac{4L^{2}}{\pi^{2}h}$.

Although the wavy asperity deviates from a spherical shape, its deformation is still small in the initial contact. Referring to the deformation of a spherical asperity, it is considered that the overall deformation of the wavy asperity in the elastic phase does not exceed the range of the small deformation (verified by the numerical analysis of the finite element), and combining with the assumptions and scope of application of the Hertz contact theory, the contact radius, contact area, and contact loads of the model are also adopted by the Hertz contact theory in the elastic phase, so the formulas of the Hertz contact theory are adopted, and the contact radius $a_{e}=\sqrt{R\omega }$, contact area $A_{e}=\pi a_{e}^{2}=\pi R\omega$, and contact load $P_{e}=\frac{4}{3}E^{*}R^{1/2}\omega^{3/2}$ are collated [2].

#### 2.1.2. Elastoplastic Phase $\left(\omega_{e}<\omega <\omega_{p}\right)$

Referring to the elastic–plastic modeling research history of previous scholars, high-order polynomials have advantages in fitting the data [7]; however, oscillations and the Runge phenomenon exist on the elastic–plastic curve; therefore, this paper chooses the excessively smooth monotonicity of the low-order function for correction. Combining the descriptions of mechanical behaviors in the elastoplastic phase given by several scholars [8,9,10] and the results of finite element analyses, the expansion of the plastic zone inside the wavy asperity leads to a nonlinear growth in the contact area, and its growth rate gradually slows as the contact depth ω increases. At the same time, the trend of the contact load changes in the elastic–plastic phase is a nonlinear increase, and the function needs to satisfy the requirements of strict monotonicity and continuity at the elastic–elastoplastic and elastoplastic–plastic critical points. To summarize, this paper chooses the simple, smooth, and monotonically increasing hyperbolic tangent function as the function description of this phase, and it extends the elastic solution $a_{e}$ to the elastic–plastic deformation range by introducing the correction term $\alpha \tanh[(\frac{\omega }{\omega_{c}}-1)/\beta ]$. According to the correction term, the contact radius of the elastic–plastic phase $a_{ep}$ is redefined as:

(5) $$a_{ep}=a_{e}\left[1+\alpha \tanh\left(\frac{\frac{\omega }{\omega_{c}}-1}{\beta }\right)\right]$$

Meanwhile, according to the axisymmetric property of the wavy asperity, the contact area is circular when the flat plate is in contact with the asperity, so the elastic–plastic contact area $A_{ep}$ is:

(6) $$A_{ep}=\pi a_{ep}^{2}=\pi a_{e}^{2}{\left[1+\alpha \tanh\left(\frac{\frac{\omega }{\omega_{c}}-1}{\beta }\right)\right]}^{2}=A_{e}{\left[1+\alpha \tanh\left(\frac{\frac{\omega }{\omega_{c}}-1}{\beta }\right)\right]}^{2}$$

where α,β are the elastic–plastic nonlinear expansion parameters, which are used to control the expansion rate of the plastic zone.

The elastic–plastic contact pressure $p_{ep}$ is also introduced on the basis of the elastic contact pressure $p_{e}$, and the correction term $\gamma \tanh\left(\frac{\frac{\omega }{\omega_{c}}-1}{\delta }\right)$ is given by:

(7) $$p_{ep}=p_{e}\left[1+\gamma \tanh\left(\frac{\frac{\omega }{\omega_{c}}-1}{\delta }\right)\right]$$

where γ,δ are elastic–plastic average contact pressure correction parameters to describe the effect of plastic deformation on the nonlinear growth of the load.

#### 2.1.3. Fully Plastic Phase $\left(\omega >\omega_{p}\right)$

When the contact depth is large, the contact region of the asperity undergoes a high degree of deformation; however, compared to the overall structure of the asperity, it is assumed that the range of deformation still belongs to the theory of small deformations (also verified by finite element analysis), so following the assumption of volume conservation, concerning the theory of geometrical projected area [16], the asperity of the contact region is compressed for the set of points of the height y≥h−ω under plastic deformation, which is brought into the equation of the surface contour:

(8) $$h\cos\left(\frac{\pi x}{2L}\right)\geq h-\omega$$

Simplifying the inequality gives:

(9) $$\cos\left(\frac{\pi x}{2L}\right)\geq 1-\frac{\omega }{h}$$

Let $s=1-\frac{\omega }{h}$; then, the inequality simplifies to:

(10) $$\cos\left(\frac{\pi x}{2L}\right)\geq s$$

where s∈[0,1].

The above cosine function cos(θ) is even in the interval [−L,L] of $\theta =\frac{\pi x}{2L}$ and takes the maximum value at x=0 and the minimum value at x=±L. The solution of the inequality cos(θ)≥s corresponds to:

(11) |θ|≤arccos(s)

Substituting $\theta =\frac{\pi x}{2L}$, the solution is obtained as:

(12) $$|x|\leq \frac{2L}{\pi }\arccos\left(1-\frac{\omega }{h}\right)$$

Then, the contact radius of the fully plastic phase $a_{p}$ is its projection in the x direction:

(13) $$a_{p}=\frac{2L}{\pi }\arccos\left(1-\frac{\omega }{h}\right)$$

When ω<<h, the above equation is expanded by the Taylor series and simplified to obtain the following equation:

(14) $$\arccos\left(1-\frac{\omega }{h}\right)\approx \sqrt{\frac{2\omega }{h}}$$

Thus, the fully plastic contact area $A_{p}$ can be obtained as:

(15) $$A_{p}=\pi a^{2}=\pi {\left[\frac{2L}{\pi }\arccos\left(1-\frac{\omega }{h}\right)\right]}^{2}\approx \frac{8L^{2}\omega }{\pi h}$$

The average contact pressure $p_{p}$ in the plastic phase can be found in Ref. [5]; when the asperity enters into fully plastic deformation, the pressure distribution in the contact area tends to be uniform, and the average value is equal to the hardness of the material H.

(16) $$p_{p}=H$$

Therefore, the contact load $P_{p}$ can be obtained:

(17) $$P_{p}=p_{p}A_{p}=H\pi {\left[\frac{2L}{\pi }\arccos\left(1-\frac{\omega }{h}\right)\right]}^{2}\approx \frac{8HL^{2}\omega }{\pi h}$$

In summary, the theoretical formulas for the wavy asperity in the three phases of elastic, elastoplastic, and fully plastic are shown in Table 1:

The above equation must satisfy the following condition, i.e., at the elastic and elastoplastic critical point, $\omega =\omega_{e}=\omega_{c}$, i.e., the continuity of contact radius, contact area, and average contact pressure, respectively, is satisfied, viz:

(18) $$a_{ep}=a_{e}\Rightarrow a_{e}\left[1+\alpha \tanh\left(\frac{\frac{\omega }{\omega_{c}}-1}{\beta }\right)\right]=a_{e}$$

(19) $$A_{ep}=A_{e}\Rightarrow A_{e}{\left[1+\alpha \tanh\left(\frac{\frac{\omega }{\omega_{c}}-1}{\beta }\right)\right]}^{2}=A_{e}$$

(20) $$p_{ep}=p_{e}\Rightarrow p_{e}\left[1+\gamma \tanh\left(\frac{\frac{\omega }{\omega_{c}}-1}{\delta }\right)\right]=p_{e}$$

The continuity condition is also satisfied at the critical elastoplastic and fully plastic point $\omega =\omega_{p}=110\omega_{c}$, i.e.,:

(21) $$a_{ep}=a_{p}\Rightarrow \sqrt{R\omega }\left[1+\alpha \tanh\left(\frac{\frac{\omega }{\omega_{c}}-1}{\beta }\right)\right]=\frac{2L}{\pi }\arccos\left(1-\frac{\omega }{h}\right)$$

(22) $$A_{ep}=A_{p}\Rightarrow \pi R\omega {\left[1+\alpha \tanh\left(\frac{\frac{\omega }{\omega_{c}}-1}{\beta }\right)\right]}^{2}=\pi {\left[\frac{2L}{\pi }\arccos\left(1-\frac{\omega }{h}\right)\right]}^{2}$$

(23) $$p_{ep}=p_{p}\Rightarrow \frac{4}{3}E^{*}R^{1/2}\omega^{3/2}\cdot \left[1+\gamma \tanh\left(\frac{\frac{\omega }{\omega_{c}}-1}{\delta }\right)\right]=H\pi {\left[\frac{2L}{\pi }\arccos\left(1-\frac{\omega }{h}\right)\right]}^{2}$$

### 2.2. Rough Surface Contact Model

In the study of rough surface normal contact of connected structures, the height distribution of the asperity can be expressed in various forms, such as the exponential distribution [17], the Weibull distribution [18], the logarithmic distribution [19], and the distribution of the MB function using the fractal model of the contact [20], etc., as well as the classical Gaussian random distribution [2,10,13], which is also one of the commonly used statistical distribution functions. In this paper, we construct an elastoplastic contact model of a wavy asperity and combine it with a Gaussian distribution [21] to establish a correspondence between the normal load on a rough surface and the average contact depth. To analyze the influence of the wavy asperity on the whole rough surface contact mechanics model and compare the difference with the spherical asperity, this paper chooses the Gaussian distribution commonly used in a variety of models for the height distribution, and its functional expression is:

(24) $$\phi (z|\mu ,\sigma^{2})=\frac{1}{\sqrt{2\pi \sigma^{2}}}e^{-\frac{{\left(z-\mu \right)}^{2}}{2\sigma^{2}}}$$

where $\sigma^{2}$ is the variance of the height distribution, and *μ* is the average height.

When the contact occurs on the rough surface, from a macroscopic overall point of view, as long as the contact surface is not completely flat, the phenomenon of partial stress concentration will inevitably occur, and the asperity will undergo elastic deformation and plastic deformation, and the contact load of all the asperities can be added up to get the amount of change in the contact state of the entire rough surface normal to the surface, i.e.:

(25) $$A_{total}=\eta \int_{d}^{d+\omega_{e}}A_{e}(\omega )\phi (z)dz+\eta \int_{d+\omega_{e}}^{d+\omega_{p}}A_{ep}(\omega )\phi (z)dz+\eta \int_{d+\omega_{p}}^{\infty }A_{p}(\omega )\phi (z)dz$$

(26) $$P_{total}=\eta \int_{d}^{d+\omega_{e}}N_{e}(\omega )\phi (z)dz+\eta \int_{d+\omega_{e}}^{d+\omega_{p}}N_{ep}(\omega )\phi (z)dz+\eta \int_{d+\omega_{p}}^{\infty }N_{p}(\omega )\phi (z)dz$$

(27) $$p_{total}=\eta \int_{d}^{d+\omega_{e}}p_{e}(\omega )\phi (z)dz+\eta \int_{d+\omega_{e}}^{d+\omega_{p}}p_{ep}(\omega )\phi (z)dz+\eta \int_{d+\omega_{p}}^{\infty }p_{p}(\omega )\phi (z)dz$$

where $A_{total}$ is the actual contact area of the rough surface, $P_{total}$ is the total contact load on the rough surface, $p_{total}$ is the average contact pressure on the rough surface, η is the number of asperities actually in contact on the rough surface, and d is the distance between the contact surface and the reference plane of the average height of the asperities.

### 2.3. Verification of Discretization of Wavy Asperity

Physical experiments of a single asperity are difficult to carry out under the current experimental conditions, and most studies have relied on the analysis of theoretical derivation and numerical simulation calculations [21]. In this paper, the same finite element method [22] (FEM) is used to carry out the discretization analysis, the ANSYS 2025 finite element software is selected to carry out the theoretical model validation, and analyses are conducted on the reasonableness of the discretization to ensure that the discretization is carried out.

#### 2.3.1. Asperity Model Construction

**Figure 1:**
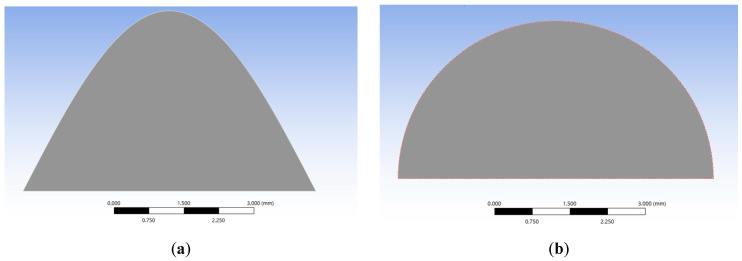
Planar model of the asperity. (**a**) Wavy asperity; (**b**) spherical asperity.

In the finite element software ANSYS 2025, wavy and spherical asperity models are established. Since both types of asperities exhibit axial symmetry in three-dimensional structures, they can be regarded as a single plane swept 180°. To simplify the calculation, the construction of the asperity is carried out in the two-dimensional plane xoy (Figure 1), and it is constrained to be axisymmetric in the z-direction. The material parameters of the asperity contact model are shown in Table 2. Mild steel is selected for the asperity, and structural steel with a yield strength slightly larger than that of the asperity is selected for the contact plate so that the contact pressure is mainly reflected in the asperity.

In order to compare and analyze the effect of the morphology of the asperity on the elastoplastic model, the wave function is set to be equal to the volume of the asperity constructed by the semicircle, the cosine asperity model $y=h\cos\left(\pi x/2L\right)$ and hemispherical asperity are established, the length of the bottom edge of the two types of asperities is 2π mm, and the areas are kept equal on the two-dimensional plane. They are summarized in Table 3.

For meshing, a high-density mesh is used in the contact area (near the vertex), which is gradually coarsened away from the contact area, and a planar constraint is set to constrain the normal displacement and a symmetric boundary to constrain the radial displacement. The bottom end of the asperity is fixed, and a normal displacement (0.01 mm) is applied to the flat plate. The contact pairs are set to have frictionless contact, while the enhanced Lagrange algorithm is chosen for calculation to avoid the error caused by penetration. The mesh adopts an adaptive algorithm, the minimum cell size is less than 1/20 of the length of the bottom edge, which is 0.03 mm, while the mesh coupling and mesh encryption are set on the contact surface of the flat plate and the asperity, the number of mesh expansion layers is set to 5, and the large deformation is turned on at the same time.

#### 2.3.2. Discretization Error Analysis

To avoid the impact of differences in calculation results caused by different degrees of discretization in finite element models on subsequent parameter fitting and analysis verification, this paper conducts mesh sensitivity tests on two types of two-dimensional models of asperity. Since the radius of curvature of the wavy asperity at the vertex is smaller compared with that of the spherical one, the variation in contact load is larger, which is more likely to lead to contact error and contact non-convergence, so it is necessary to analyze the error of the wavy asperity. The size of the grid cell is set to 0.03 mm, and the size of the grid cell is set to 0.01 mm, respectively, to determine whether the 0.03 mm grid quality meets the contact convergence conditions, and by applying the same vertical downward displacement load to the flat plate, comparing the difference between the equivalent stresses and strains, and calculating the size of the error, to analyze whether it can satisfy the computational needs of discretization, and the result can be analogous to that of the discretization of the spherical asperity problem.

**Figure 2:**
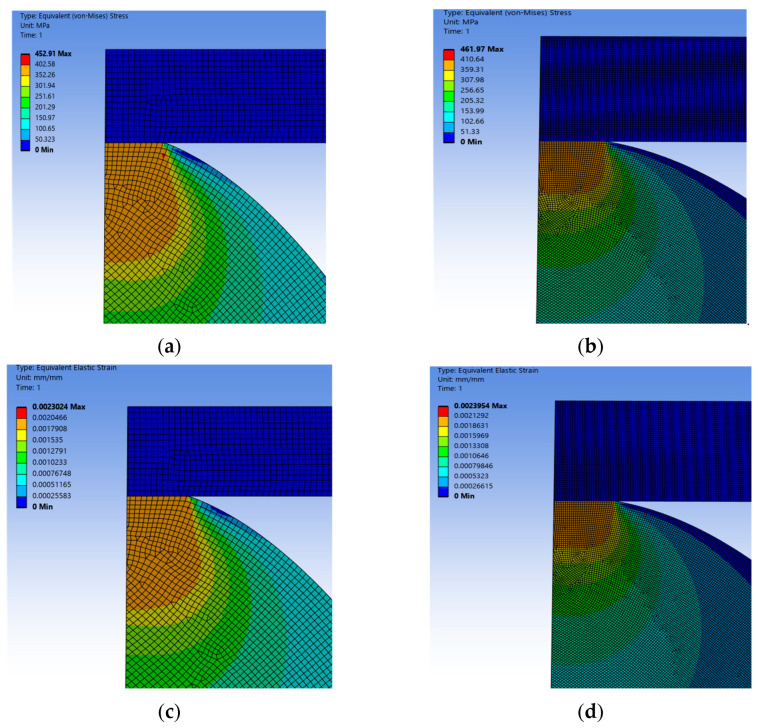
Mesh refinement of the wavy asperity model. (**a**) Mesh density 0.03 mm equivalent stress; (**b**) mesh density 0.01 mm equivalent stress; (**c**) mesh density 0.03 mm equivalent strain; (**d**) mesh density 0.01 mm equivalent strain.

According to the mesh quality inspection and normal displacement of the asperity under an equivalent force and equivalent strain, the results are shown in Figure 2:

According to the grid sensitivity test (Table 4), the total number of cells increases nearly 10 times when the overall discretized grid size is reduced from 0.03 mm to 0.01 mm, and at the same time, the encrypted mesh quality meets the numerical analysis grid quality requirements, judging from the cell quality and the Jacobi coefficient, which are both close to 1.0.

From the error analysis of equivalent strain and equivalent force, the errors of stress and strain of the two sizes of mesh are 3.8% and 1.9%, respectively, which are all within the allowable 5% error of the finite element, so the lower density mesh can meet the numerical analysis requirements of the discretization of the asperity and the real mechanical response.

According to the calculation results after mesh refinement, there is a certain improvement compared with the original mesh, which indicates that the numerical calculation results of the asperity with a lower degree of discretization are more conservative compared with the real asperity’s contact state, which is in line with the objective physical law, and the calculation error is within the acceptable range, so it can be continued to carry out the subsequent calculations and analyses.

### 2.4. Validation of Contact Model of Wavy Asperity

#### 2.4.1. Stress–Strain Distribution

**Figure 3:**
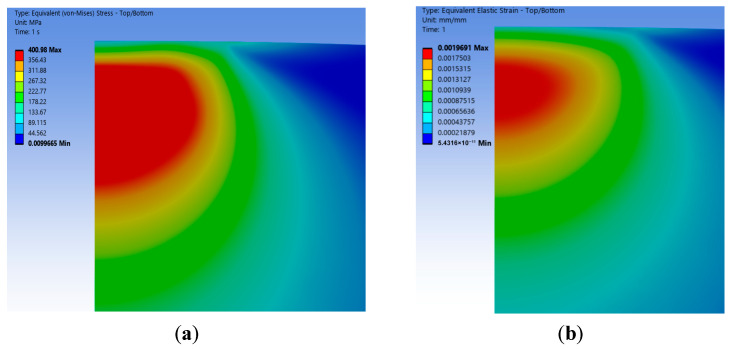

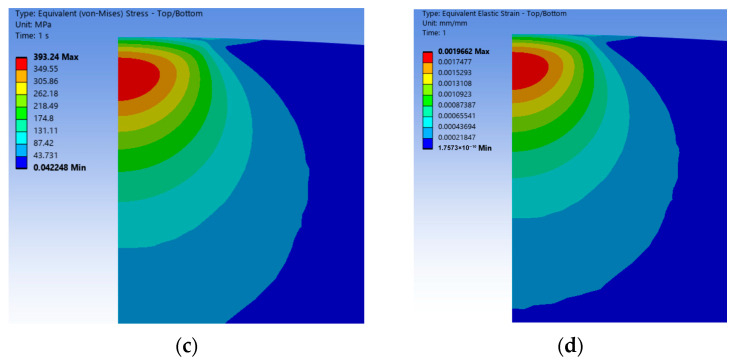
Equivalent stress and strain nephogram in elastic phase $\left(\omega =\omega_{c}\right)$. (**a**) Wavy equivalent stress; (**b**) wavy equivalent elastic strain; (**c**) spherical equivalent stress; (**d**) spherical equivalent elastic strain.

According to the geometrical and numerical calculations of the discretized model, the equivalent stress and equivalent strain distributions of the two types of asperities under the same normal displacement show significant differences. Firstly, the distribution characteristics of the stress and strain in the elastic phase $\left(\omega \leq \omega_{e}\right)$ are analyzed as follows (Figure 3a–d):

(1) The equivalent stress value of the wavy asperity is 400.98 MPa, which is close to the yield strength limit of the asperity, and the stress size and distribution are in line with the real contact surface; observing the stress and strain distribution area, it is mainly concentrated in the downward position of the contact surface, presenting non-elliptic symmetry and showing nonlinear increasing and then decreasing changes along the axial y direction. Combined with the fact that the contact area is much smaller than the radius of the bottom surface R, which is still in the range of small deformation, it proves that the Hertz theory is also applicable to the wavy asperity.
(2) According to the classic Hertz theory prediction, the maximum stress distribution of a spherical asperity is also concentrated at the lower position of the contact surface, and the stress concentration zone is closer to the bottom than that of the wavy asperity, with a stress value of 393.24 MPa, and it is axisymmetric and semi-ellipsoidal in distribution. In terms of numerical values, the difference between the spherical asperity and the specified material yield strength is greater than that of the wavy asperity, and the strain is also relatively small compared to the wavy asperity (0.00196691 mm). It is believed that based on Hertz’s elastic theory, the radius of curvature of the wavy asperity is 33% of that of the spherical one, which leads to a more centralized stress in the wavy asperity, and thus, the stress–strain distribution of the wavy asperity in the elastic phase is more accurate than that of the spherical one.

**Figure 4:**
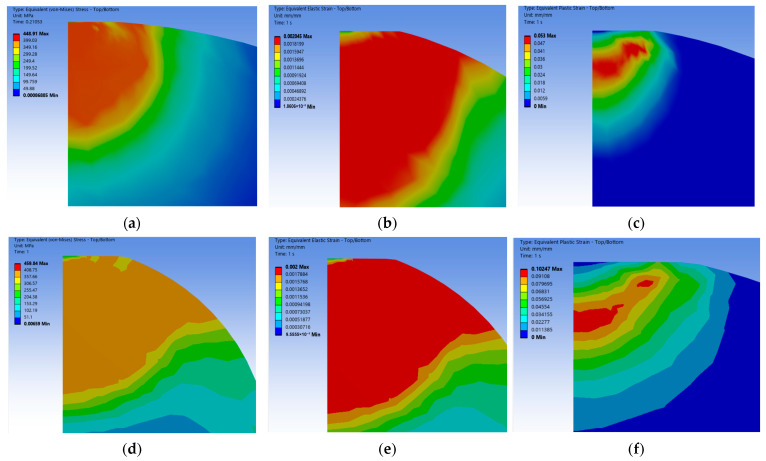
Equivalent stress and strain nephogram in elastoplastic phase $\left(\omega =50\omega_{c}\right)$. (**a**) Wavy equivalent stress; (**b**) wavy equivalent elastic strain; (**c**) wavy equivalent plastic strain; (**d**) spherical equivalent stress; (**e**) spherical equivalent elastic strain; (**f**) spherical equivalent plastic strain.

According to the stress–strain of wavy and spherical asperities in the elastoplastic phase $\left(\omega_{e}\leq \omega \leq \omega_{p}\right)$, see Figure 4a–f, the stress and strain distribution laws are analyzed in terms of the elastoplastic behavior of the asperity and the respective growth laws of the two types of asperities:

(1) The equivalent stresses of the wavy asperity and the spherical asperity are closer to each other in the elastoplastic phase, and they jointly show that the stress in the elastic phase rises slightly with the increase in the contact depth, but the overall change is kept flat; in contrast, the upward trend of the plastic strain varies more, showing a rapid increase in the elastoplastic beginning, and then the upward trend tends to flatten out. The whole elastoplastic change behavior can conform to the predicted values of the elastoplastic model of ZMC [6] and Brake [9] et al.
(2) The equivalent elastic strains of the wavy asperity and the spherical asperity at this phase are roughly similar in terms of the numerical values and the strain distribution cloud diagrams, with the elastic strains being around 0.002 mm, while the plastic strain of the wavy asperity is 0.0532 mm, which is about 52% of that of the spherical asperity, suggesting that the increase in plastic strain of the wavy asperity slows down with the increase in the depth of contact. Whereas the spherical asperity is a purely ideal case, the curvature does not change, which means that the plastic deformation of the spherical asperity is more rapid, and the trend of the stiffness is not as obvious as that of the wavy asperity. In the strain distribution region, the overall strain distribution of the wavy asperity shows asymmetric plastic flow (Figure 4c), with localized shear zones appearing at the contact edges. According to Chu et al.’s [13] indentation experiments, the real surface activates multiple localized contact points under load, which means that the real surface morphology generates a complex multiaxial stress state, and thus, the strain concentration phenomenon is more pronounced in the contacted individual asperity. While the single point of contact predicted by the spherical model (Hertz theory) is not consistent with the actual observation, the change in the wavy asperity in the elastoplastic phase is closer to the real physical mechanism.

**Figure 5:**
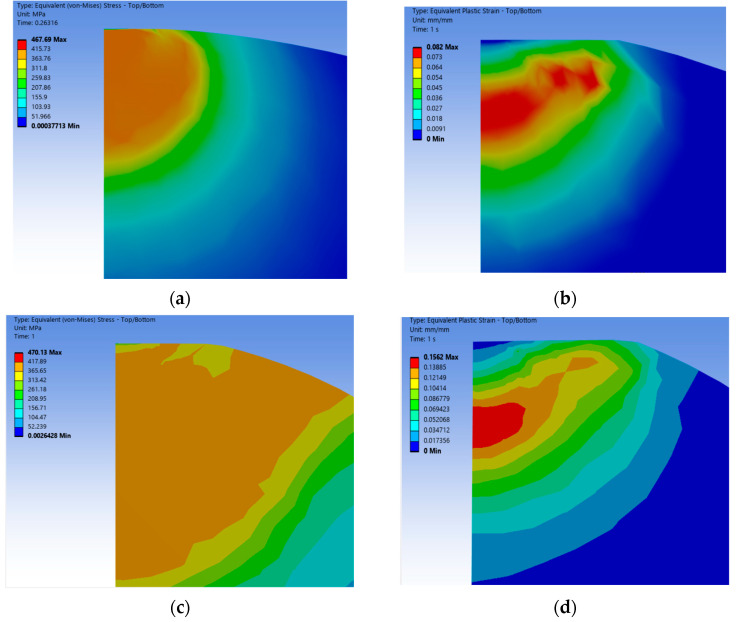
Equivalent stress and strain nephogram in plastic phase $\left(\omega =110\omega_{c}\right)$. (**a**) Wavy equivalent stress; (**b**) wavy equivalent plastic strain; (**c**) spherical equivalent stress; (**d**) spherical equivalent plastic strain.

For the stress–strain of the wavy asperity and the spherical asperity in the fully plastic phase $\left(\omega >\omega_{p}\right)$, see Figure 5a–d. The following is an analysis of the patterns of change.

The equivalent stress distribution of the wavy asperity is similar to that of the sphere, which is 467.69 MPa and 470.13 MPa, respectively, the wavy asperity stress is 99.5% of the sphere, both of which are close to the hemispherical downward expansion tendency, the equivalent plastic strain of the wavy asperity is 0.082 mm, that of the sphere is 0.1562 mm, and the ratio of strain is close to 52%, indicating that in the elastoplastic near-plastic phase, the wavy and spherical plastic strains are close to the same growth trend. In this phase, the strain distribution of the wavy asperity also exhibits asymmetric plastic flow. The plastic changes near the contact surface are relatively gradual, while those of the spherical asperity are more concentrated in the axial region. In comparison with the physical mechanism of elastoplastic mechanics, the wavy asperity is more consistent with actual conditions.

#### 2.4.2. Elastoplastic Contact Model

In the process of discrete numerical calculation, to analyze how the contact stress of wavy asperity contact increases with time (contact depth), the change rule of the contact area, contact load, and average contact pressure, identify the correction parameters, and obtain a more accurate elastoplastic contact model, the finite element numerical results are processed accordingly, and the key conclusions are obtained.

Table 5 is shown below.

Meanwhile, concerning the elastic and elastoplastic objective functions, the empirical formulas were matched with the finite element data, and the least squares method was used to minimize the residual sum of squares:

(28) $$\frac{a_{ep}}{a_{e}}=1+\alpha \tanh\left(\frac{\frac{\omega }{\omega_{c}}-1}{\beta }\right);\;\underset{\alpha ,\beta }{\min}{\sum \left(\frac{a_{ep}^{FEM}}{a_{e}}-\left[1+\alpha \tanh\left(\frac{\frac{\omega }{\omega_{c}}-1}{\beta }\right)\right]\right)}^{2}$$

(29) $$\frac{A_{ep}}{A_{e}}={\left[1+\alpha \tanh\left(\frac{\frac{\omega }{\omega_{c}}-1}{\beta }\right)\right]}^{2};\;\underset{\alpha ,\beta }{\min}{\sum \left(\frac{A_{ep}^{FEM}}{A_{e}}-{\left[1+\alpha \tanh\left(\frac{\frac{\omega }{\omega_{c}}-1}{\beta }\right)\right]}^{2}\right)}^{2}$$

(30) $$\frac{p_{ep}}{p_{e}}=1+\gamma \tanh\left(\frac{\frac{\omega }{\omega_{c}}-1}{\delta }\right);\;\underset{\gamma ,\delta }{\min}{\sum \left(\frac{p_{ep}^{FEM}}{p_{e}}-\left[1+\gamma \tanh\left(\frac{\frac{\omega }{\omega_{c}}-1}{\delta }\right)\right]\right)}^{2}$$

**Figure 6:**
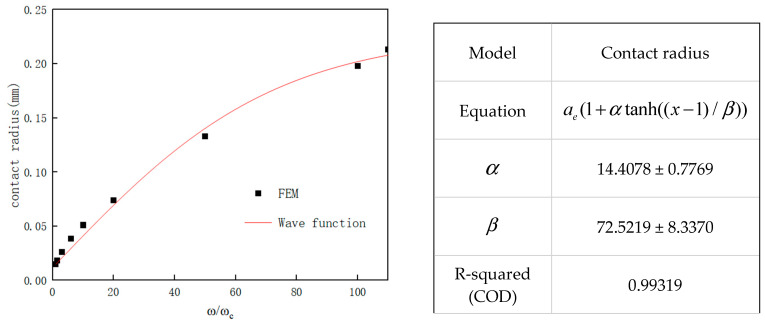

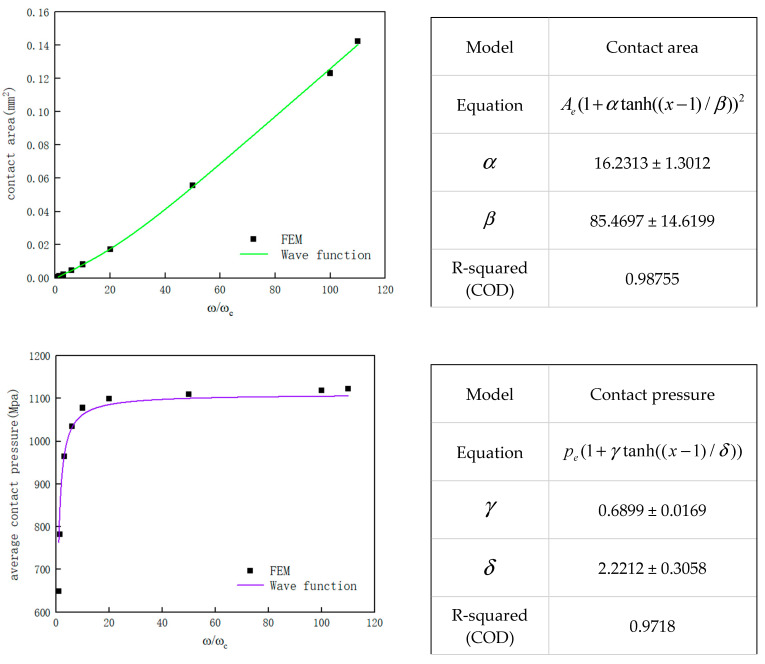
Identification of correction parameters of elastic–plastic function.

The parameters and fitting equations of the wavy asperity were imported into MATLAB 2022 for nonlinear fitting, and the relevant data and curves of the contact radius, contact area, and contact load were obtained, as shown in Figure 6:

According to the finite element numerical results and theoretical formulas for fitting the correction parameters, the optimization coefficients α≈15.4, β≈80.8, γ≈0.73, and δ≈2.20 are obtained for the contact radius, contact area, and average contact pressure.

In this paper, the key parameters of the correction term of the hyperbolic tangent function are obtained through the fitting of the finite element numerical analysis results and theoretical formulas, the elastoplastic theoretical model of the wavy asperity is determined, and the differences between the mechanical behavior of the model and the spherical asperity as well as the degree of agreement with the actual physical laws are analyzed through the finite element numerical analysis results of the asperity of the two shapes, as shown in Table 5 as well as Table 6:

(1) Elastic phase $\left(\omega /\omega_{c}\leq 1\right)$: The contact area and load of the wavy asperity coincide with the results of the Hertz contact theory, while the initial contact area of the wavy asperity is smaller than that of the sphere due to the smaller radius of curvature of the contact vertices, which is 33% of that of the sphere, and the errors between the finite element values of both of them and the theoretical formulas are within 5%, which indicates that the use of the Hertz contact theory is correct in the elastic phase. The Hertz contact theory is correct and applicable to both types of asperities.
(2) Elastic–plastic phase $\left(1<\omega /\omega_{c}\leq 110\right)$: At the initial phase of $\omega /\omega_{c}=1.5$, the contact area of the wavy asperity grows at a multiplier of about 1.83, and that of the spherical body is about 1.5, and the growth rate is about 22% faster than that of the spherical body. The growth rate of the contact load of the wavy body grows at a multiplier of about 1.89, and that of the spherical body is about 1.8, and the growth rate is similar. The contact pressure growth multiplier is about 1.2 for the wavy asperity and about 1.23 for the spherical body, and the growth rate is about 3% slower than that of spherical body because in this phase, the radius of curvature of the wavy asperity is small, which leads to the fast diffusion of the plastic region; while reaching the plastic phase at $\omega /\omega_{c}=100$, the contact area, contact load, and average growth rate of the contact pressure of the wavy asperity and the spherical body converge to the same level, and the theoretical and numerical results converge to the same level. This indicates that the mechanical behaviors of the wavy and spherical asperities show monotonically increasing trends throughout the elastoplastic phase, but the increasing trend of the wavy asperity slows down, which is consistent with the conclusion that there is a critical point in the KE model in the elastoplastic phase.
(3) Fully plastic phase $\left(\omega /\omega_{c}>110\right)$: The contact area of the wavy asperity follows the projected area theory, and the flattening process of the numerically analyzed asperity is consistent, whereas the spherical model overestimates the contact area by nearly 20% due to the assumption of volume conservation (1.1014 mm^2^ versus 1.3028 mm^2^, calculated by $A_{p}=2\pi R\omega$), which is consistent with the experimental situation in Ref. [13], and in terms of the contact radius, the contact radius is 18.8% of the radius of the asperity. It is debatable whether the small deformation assumption can be continued, so the wavy asperity is more consistent with the real contact situation than the spherical asperity.

On the other hand, in order to verify the continuity of the improved model in this paper and whether there is any oscillation phenomenon, the model in this paper is compared with the classical models of scholars in recent years, respectively, and analyzed around various types of elastoplastic improved asperity models. To make the comparison more obvious, the regularization method is used to normalize the main contact parameters, and the normal contact depth is normalized to:

(31) $$\omega^{*}=\frac{\omega }{\omega_{c}}$$

The asperity contact state variables are normalized as:

(32) $$A^{*}=\frac{A}{A_{e}}=\frac{A}{\pi R\omega_{c}}$$

(33) $$P^{*}=\frac{P}{P_{e}}=\frac{P}{\frac{4}{3}E^{*}R^{1/2}\omega^{3/2}}$$

(34) $$p^{*}=\frac{p}{H}$$

The above normalization covers the whole phase, from elasticity to full plasticity.

**Figure 7:**
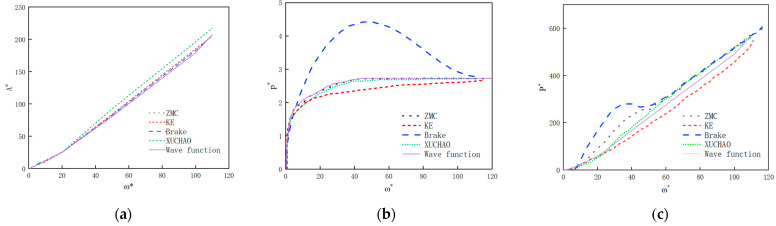
Comparison of contact characteristics of asperities with different statistical models. (**a**) Normalized contact area; (**b**) normalized average contact pressure; (**c**) normalized contact load.

**Figure 8:**
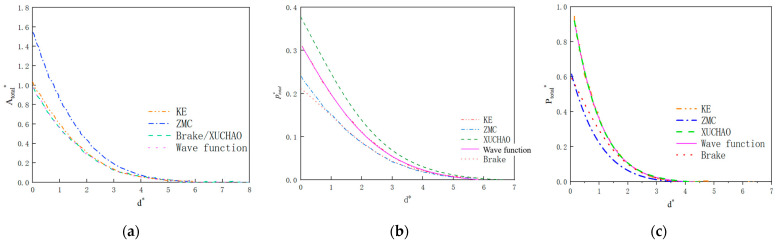
Rough surface normal contact model. (**a**) Normalized actual contact area; (**b**) normalized actual contact pressure; (**c**) normalized actual contact load.

In comparison with the contact properties of other statistical summation models, the details are shown in Table 7, as follows:

As shown in Figure 7, the contact characteristic curves predicted by the five models in Table 7 all satisfy continuity at the critical point. However, the use of higher-order interpolation polynomials can lead to changes in the predicted curves that do not conform to the elastic–plastic law. Specifically, as shown in Figure 8b, the average contact pressure predicted by the Brake model exhibits non-monotonic changes, which do not align with the physical principles of contact mechanics. Additionally, at the elastic–elastoplastic and elastoplastic–plastic transition points, there is also a noticeable Runge oscillation phenomenon. Furthermore, the contact load predicted by the KE model and Brake model in Figure 8c exhibits a trend of first increasing and then decreasing. Comparison reveals that the load curves predicted by the ZMC model, Xu model, and the model proposed in this paper all satisfy continuity and monotonicity. However, due to differences in interpolation methods, interpolation polynomial-based models exhibit non-uniqueness. The hyperbolic tangent correction model adopted in this study achieves an error of <5% when fitting finite element discretization data, with a smoother elastic–plastic curve, resolving the non-physical oscillation issues in the KE and Brake models and featuring a simpler overall expression.

#### 2.4.3. Surface Contact Model

The rough surface contact state variables *A_total_*, *P_total_*, and *p_total_* are normalized:

(35) $$A_{total}^{*}=\frac{A_{total}}{\eta \pi R\omega_{c}}$$

(36) $$P_{total}^{*}=\frac{P_{total}}{\eta \frac{4}{3}E^{*}R^{1/2}\omega_{c}^{3/2}}$$

(37) $$p_{total}^{*}=\frac{p_{total}}{\eta H}$$

(38) $$d^{*}=\frac{d}{\sigma }$$

where d is the distance between the rough surface and the average height of the asperity. Figure 8 gives a comparison between the rough surface contact model of this paper and the other models. With the increase in normal proximity, the distance between the average height of the elastic flat plate and the asperity decreases gradually, and the actual contact area, the average contact pressure, and the total normalized contact load all show a nonlinear growth. The prediction results of this paper’s model and the other four models are consistent in the overall rule of change, which indicates that this paper’s model is also applicable to the physical mechanism of contact mechanics.

With the increase in contact depth, the number of asperities undergoing plastic deformation increases, and the relationship between the contact state variables predicted by the various models and the change in the average contact depth appears to be different. In terms of the trend of the actual contact area, the models in this paper and the KE model use the analytical solution of the discretized numerical analysis, so the predictions are close to each other, and compared with the traditional spherical asperity surface, the wavy asperity elastoplastic model of this paper has a more gentle change. In terms of the actual contact pressure, the wavy asperity of this paper is larger in the initial elastic–elastoplastic phase compared with the models of KE, ZMC, etc., which proves that the radius of curvature of the wavy asperity is smaller than that of the spherical asperity, and the contact pressure is larger due to the small initial contact area, which is in line with the actual situation. From the view of the actual contact load, the models of this paper, KE, and Xu Chao have higher actual contact load in the elastic phase compared with the conventional spherical asperity, and the results are similar in the near-plastic phase, which indicates that the change in normal contact stiffness in the wavy asperity is more obvious compared with that of the spherical asperity. The results are similar near the plastic phase, indicating that the change in normal contact stiffness of wavy asperity is more obvious compared to the spherical one.

In conclusion, the wave asperity model constructed in this paper is consistent with the laws of change in contact behavior in terms of continuity, while the change trend is more in line with the state that the increase in actual contact stiffness decreases gradually compared with the spherical asperity [23]. The difference mainly lies in the mechanical behavior of the early elastic–elastoplastic phase, which is also in line with the transformation relationship of the material from linear elasticity to nonlinearity.

**Figure 9:**
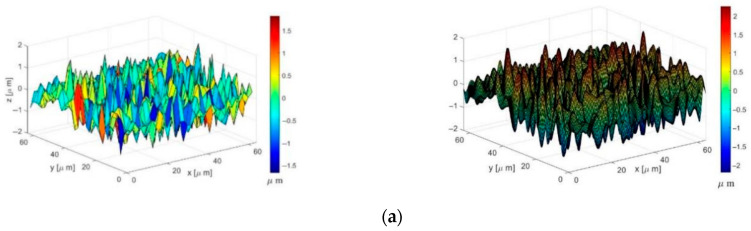

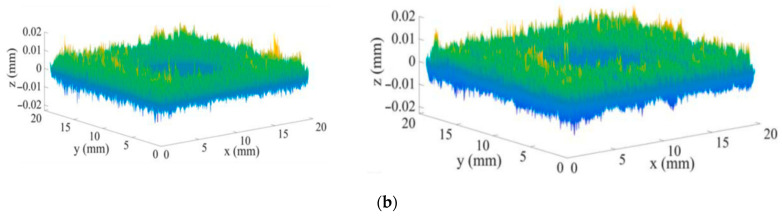
Measurement of surface morphology of rough surfaces at different scales. (**a**) Micrometer-scale rough surface morphology; (**b**) millimeter-scale rough surface morphology.

According to the above analysis of the rough surface normal contact model, the plastic flow and stress concentration phenomenon of the wavy asperity is more obvious than that of the spherical asperity, and the trend of stress–strain growth tends to flatten out with the increase in the contact depth from elastic–plastic to plastic; at the same time, from the surface topography measurement experiments of the rough surface, see Figure 9, the actual rough surface shows multi-scale periodic fluctuations, while the spherical asperity is unable to describe these asymmetric peaks and valleys due to the constant curvature; the wavy asperity can more flexibly match the power spectral density (PSD) of the measured contour by adjusting the wavelength (L) and amplitude (h). Therefore, this paper concludes that the wavy asperity has advantages over the spherical asperity in terms of the mechanical properties of the individual models and the feedback mechanism of the entire rough surface morphology. In subsequent research, wavy asperities can be prioritized [24,25].

## 3. Results

In this paper, the elastoplastic contact theoretical model of a wavy asperity is established, which contains three phases of elastic, elastoplastic, and fully plastic, and it is systematically verified by combining with discretized finite element numerical analysis. The situation is summarized as follows:

(1) This paper uses the finite element method to compare the stress–strain conditions of wavy and spherical asperities through numerical analysis. Under the assumption that discrete interference is excluded, we find that the wavy asperity exhibits asymmetric plastic flow and multiple stress concentration zones, with local shear force bands appearing at the contact edges. This is consistent with experimental observations of multi-axial stress states in real contact. Furthermore, under the same initial displacement load conditions, the behavior at $\omega =\omega_{c}$ and $\omega =110\omega_{c}$ is more consistent with contact laws than that of a spherical body.
(2) The theoretical models of the wavy asperity in the elastic, elastoplastic, and fully plastic phases are derived. The Hertz contact theory is adopted in the elastic phase, and the radius of curvature at the apex of the wavy asperity is $R\propto \frac{L^{2}}{h}$, which is 33% of that of the spherical asperity of the equal bottom edge length, leading to a more centralized initial contact stress; in the elastoplastic phase, the hyperbolic tangent function (tanh) is introduced to describe the nonlinear transition, which ensures the continuity and monotonicity of the critical points ($\omega =\omega_{e}$ and $\omega =\omega_{p}$). Combining finite element analysis and least squares fitting, the parameters α=15.4, β=80.8, γ=0.73, and δ=2.20 are obtained, and the goodness-of-fit is more than 0.97. In the fully plastic phase, it follows the geometric projection theory ($A_{p}\propto \frac{L^{2}\omega }{h}$), which avoids the overestimation of the 20% area of the spherical asperity caused by the assumption of the conservation of volume.
(3) Using the classical Gaussian distribution as the height distribution of the asperity, a rough surface contact model with a wave function is derived, whose contact stiffness changes are more obvious than those of the spherical shape in the initial elastic–plastic phase, and the overall contact behavior is more gentle. Combined with the rough surface topography mapping experiments, the wavy asperity can better describe the multi-peak topography and the feedback mechanism of alternating peaks and valleys by adjusting the parameters (L and h), which is superior to the spherical asperity in terms of power spectral density.

## 4. Discussion

The authors compare the model in this paper with several classic models in terms of core assumption differences, key performance indicators of the asperity elastic–plastic model, and applicable scenarios. From the perspective of the model’s mechanism, the advantages of the model in this paper are more obvious, as shown in Table 8 below:

Combining the physical mechanisms of several classic models, this paper conducts a more precise analysis according to theoretical derivations and finite element simulation results using the model presented herein. The new wavy asperity model constructed using the cosine function proposed in this paper breaks through the geometric assumptions of traditional spherical asperity models. It has certain advantages in the theoretical derivation of the elastoplastic curve of a single asperity, the comparison and verification of finite element numerical analysis results, and the contact characteristics of rough surfaces composed of multiple asperities. Specifically, these advantages are manifested as follows:

(1) Differences in mechanical response of asperity

Authenticity of stress concentration: The curvature radius of the wavy asperity is smaller in the initial phase of elastic-to-elastoplastic deformation, leading to higher stress concentration (Figure 3a). Therefore, the phenomenon of plastic deformation is more pronounced compared to the spherical asperity (Figure 4c,f), resulting in asymmetric plastic flow and local shear bands (Figure 4c and Figure 5b), consistent with experimental observations under multi-axial stress conditions [13]. As contact depth increases, the rate of increase in contact radius for the wavy asperity rapidly decreases (Figure 7), enabling it to transition to the fully plastic phase of mechanical behavior more quickly than the spherical asperity.

Accuracy of mechanical behavior: Using the Hertz contact theory in the elastic phase, the contact area growth rate in the elastic phase is 22% higher than that in the spherical asperity. The stress at the critical point of the elastic-to-elastoplastic phase is closer to the yield strength of the material. In the elastoplastic phase, the correction term of the tanh function is used to eliminate non-physical oscillations and the Runge phenomenon caused by high-order polynomials of the ZMC model and Brake model, solve the key defects of the elastoplastic transition model, and achieve smooth transition at $\omega =\omega_{c}$ and $\omega =110\omega_{c}$ (Figure 7). The fitting of the correction parameters (α,β,γ,δ) in the elastoplastic phase to the finite element simulation results is R-squared (COD) = 0.97, and the robustness of the model is verified. In the plastic phase, the projected area theory is selected to correct the errors in the contact area of the volume conservation assumption in the other four models during the plastic stage. The wavy asperity exhibits a trend of rapid increase followed by gradual saturation in the contact area, contact load, and contact pressure curves throughout the elastic-to-fully plastic phase, which effectively reflects the nonlinear hardening behavior of the metal material’s stiffness.

(2) Geometric characteristics of rough surfaces

Asymmetry and geometric diversity: Spherical asperities cannot adjust their contact radius due to geometric simplification, while wavy asperities of the same volume have a smaller curvature radius and can be adjusted by regulating the wavelength (L) and amplitude (h). When combined with the power spectral density distribution (Figure 9), this enables a more precise description of the sharpness of rough surfaces and the total number of asperities, overcoming the limitations of spherical symmetry and ensuring the geometric diversity of rough surfaces.

Contact density: The spherical asperity underestimates the contact point density due to geometric simplification. The wavy asperity, with a smaller curvature radius (33% of that of spherical ones), can increase the initial number of contact points and lead to higher stress concentration, consistent with experimental phenomena involving interactions between multiple asperities.

## 5. Conclusions

This paper employs a combination of theoretical derivation and finite element numerical analysis for verification, establishing a new elastic–plastic contact model for a wavy asperity. This model unifies the elastic–plastic theoretical framework, integrating the Hertz contact theory, the modified tanh elastoplastic transition function, and the projected area theory, achieving seamless transitions between elastic, elastoplastic, and fully plastic phases. This model satisfies the continuity and monotonicity requirements of a single-asperity elastoplastic contact model, better aligning with mechanical principles compared to spherical asperities and offering greater accuracy and intuitiveness than other models. The modified parameters can be rapidly identified using the least squares method based on finite element analysis results.

Additionally, the wavy asperities constructed in this paper can associate the geometric characteristics of rough surfaces with physical mechanisms. By modeling the height distribution of asperities that matches the power spectral density, the model demonstrates the asymmetric stress distribution and multi-zone plasticity mechanical characteristics of real rough surface contacts. Since the function of the asperity height distribution is not the focus of this study, the classical Gaussian distribution was selected. However, recent research suggests that fractal models such as MB and WM may better characterize the height distribution characteristics of asperities on rough surfaces. Future studies could incorporate experimental research to more precisely correlate the mechanical behavior of microscopic asperities with the mechanical behavior of macroscopic contact surfaces.

## Figures and Tables

**Table 1:** Elastoplastic theoretical model of wavy asperity.

Phase	Contact Radius a	Contact Area A	Contact Load P	Average Contact Pressure p
Elastic	$a_{e}=\sqrt{R\omega }$	$A_{e}=\pi R\omega$	$P_{e}=\frac{4}{3}E^{*}R^{1/2}\omega^{3/2}$	$p_{e}=\frac{4}{3\pi }E^{*}R^{-1/2}\omega^{1/2}$
Elastoplastic	$a_{ep}=a_{e}\left[1+\alpha \tanh\left(\frac{\frac{\omega }{\omega_{c}}-1}{\beta }\right)\right]$	$A_{ep}=A_{e}{\left[1+\alpha \tanh\left(\frac{\frac{\omega }{\omega_{c}}-1}{\beta }\right)\right]}^{2}$	$P_{ep}=A_{ep}p_{ep}$	$p_{ep}=p_{e}\left[1+\gamma \tanh\left(\frac{\frac{\omega }{\omega_{c}}-1}{\delta }\right)\right]$
Fully plastic	$a_{p}\;\approx \frac{2L}{\pi }\sqrt{\frac{2\omega }{h}}$	$A_{p}\approx \frac{8L^{2}\omega }{\pi h}$	$P_{p}=HA_{p}\approx \frac{8HL^{2}\omega }{\pi h}$	$p_{p}=H$

**Table 2:** Material parameters of asperity.

Parameter Type	Modulus of Elasticity E(GPa)	Poisson’s Ratio ν	Yield Strength Y (MPa)	Hardness H
Asperity	200	0.3	400	2.8Y
Flat plate	200	0.3	460	2.8Y

**Table 3:** Interface morphology of two types of asperities.

Shape	Bottom Edge Length(mm)	Area (mm^2^)	Functional Expression
Wave function	2π	$\frac{\pi^{3}}{2}$	$y=\frac{\pi^{3}}{8}\cos\left(\frac{x}{2}\right)\;[-\pi ,\pi ]$
Semicircular			$x^{2}+y^{2}=\pi^{2}\;[-\pi ,\pi ]$

**Table 4:** Grid sensitivity analysis.

Mesh Size (mm)	Number of Grid Cells	Cell Mass	Jacobi Coefficient	Equivalent Strain (mm)	Equivalent Stress (MPa)
0.03	20,400	0.98557	0.96918	0.0023024	452.91
0.01	190,334	0.98872	0.99073	0.0023954	461.97

**Table 5:** Elastic–plastic phase of wavy asperity discretized model data.

$\mathrm{Contact}\;\mathrm{Depth}\;\omega /\omega_{c}$	Wavy Asperity			
	Contact Radius a (mm)	Contact area A (mm^2^)	Contact Load P (N)	Average Contact Pressure p (MPa)
1	0.0148	0.0006	0.42	648.86
1.5	0.0183	0.0011	0.87	781.83
3	0.0260	0.0021	2.05	965.02
6	0.0384	0.0046	4.79	1034.25
10	0.0510	0.0082	8.81	1077.87
20	0.0740	0.0172	20.47	1099.12
50	01551	0.0756	83.94	1110.35
100	0.1980	0.1236	124.25	1119.22
110	0.2130	0.1425	160.06	1123.17

**Table 6:** Discretized model data for the elastic–plastic phase of the spherical asperity.

$\mathrm{Contact}\;\mathrm{Depth}\;\omega /\omega_{c}$	Spherical Asperity			
	Contact Radius a (mm)	Contact Area A (mm^2^)	Contact Load P (N)	Average Contact Pressure p (MPa)
1	0.0371	0.0043	3.1276	723.3
1.5	0.0450	0.0064	5.6823	893.2
3	0.0635	0.0127	13.4277	1060.0
6	0.0924	0.0268	29.9309	1115.9
10	0.1430	0.0642	72.1699	1123.4
20	0.2011	0.1270	143.5409	1129.8
50	0.3731	0.4373	495.7909	1133.7
100	0.5450	0.9331	1060.2241	1136.2
110	0.5921	1.1014	1253.9292	1138.5

**Table 7:** Comparison with other statistical summation models.

Model	Contact Properties of Asperity			Critical Point		
	Contact Area	Average Contact Pressure	Contact Load	Yield	Elasticity	Full Plasticity
ZMC	4th-degree polynomial	Logarithmic polynomial	Product of contact area and average contact pressure	$\omega_{c}$	$\omega_{c}$	$54\omega_{c}$
KE	Segmental function	Exponential function			$6\omega_{c}$	$110\omega_{c}$
Brake	3rd-degree polynomial	3rd-degree polynomial			$\omega_{c}$	$110\omega_{c}$
XU CHAO	Brake model	Elliptic curve			$\omega_{c}$	$110\omega_{c}$
Wave function	Hyperbolic tangent	Hyperbolic tangent			$\omega_{c}$	$110\omega_{c}$

**Table 8:** Advantages and disadvantages of several models.

Model	Geometric Assumptions	Elastic-Plastic Transfer Function	Continuity	Oscillation Issues	Applicable Scenarios
ZMC	spherical	Higher-order polynomial	Partially satisfied	Serious (Runge)	Small deformation
KE	spherical	Piecewise function	Satisfied	Minor	Moderate plasticity
Brake	spherical	Hermite interpolation	Satisfied	Serious	Theoretical analysis
Xu Chao	spherical	Elliptic curve	Satisfied	None	Moderate deformation
Wave function	wavy	Hyperbolic tangent function	Strictly complies with	None	Large deformation/ multiscale

## Data Availability

The original contributions presented in this study are included in the article. Further inquiries can be directed to the corresponding author.
